# E4 Transcription Factor 1 (E4F1) Regulates Sertoli Cell Proliferation and Fertility in Mice

**DOI:** 10.3390/ani10091691

**Published:** 2020-09-18

**Authors:** Rong-Ge Yan, Qi-Lin Yang, Qi-En Yang

**Affiliations:** 1 Key Laboratory of Adaptation and Evolution of Plateau Biota, Northwest Institute of Plateau Biology, Chinese Academy of Sciences, Xining 810000, China; yanrongge@nwipb.cas.cn; 2 College of Life Science, University of Chinese Academy of Sciences, Beijing 100049, China; 3 Department of Veterinary Sciences, Qinghai Vocational Technical College of Animal Husbandry and Veterinary, Xining 810016, China; yangqi_lin123@163.com; 4 Qinghai Key Laboratory of Animal Ecological Genomics, Northwest Institute of Plateau Biology, Chinese Academy of Sciences, Xining 810001, China

**Keywords:** E4F1, sertoli cells, spermatogenesis, proliferation, fertility

## Abstract

**Simple Summary:**

Male fertility relies on the generation of functional sperm in seminiferous tubules of the testis. In mammals, Sertoli cells are the only somatic cells that directly interact with spermatogenic cells. Compelling evidences suggest that the number of Sertoli cells determines testis size and sperm output, however, molecular mechanisms regulating Sertoli cell proliferation and maturation are not well-understood. Using a Sertoli cell specific loss-of-function approach, here we showed that transcription factor E4F1 played an important role in murine Sertoli cell proliferation. Compared with their littermate control, *E4f1* conditional knockout male mice sired a significantly low number of pups. *E4f1* deletion resulted in reduced Sertoli cell number and testis size. Further analyses revealed that *E4f1* deletion affected Sertoli cell proliferation in the neonatal testis and caused an increase in apoptosis of spermatogenic cells without affecting normal development of spermatogonia, meiotic and post-meiotic germ cells. These findings have shed new light on molecular controlling of spermatogenesis in mice and a similar mechanism likely exists in other animals.

**Abstract:**

In the mammalian testes, Sertoli cells are the only somatic cells in the seminiferous tubules that provide structural, nutritional and regulatory support for developing spermatogenic cells. Sertoli cells only proliferate during the fetal and neonatal periods and enter a quiescent state after puberty. Functional evidences suggest that the size of Sertoli cell population determines sperm production and fertility. However, factors that direct Sertoli cell proliferation and maturation are not fully understood. Transcription factor E4F1 is a multifunctional protein that serves essential roles in cell fate decisions and because it interacts with pRB, a master regulator of Sertoli cell function, we hypothesized that E4F1 may have a functional role in Sertoli cells. *E4f1* mRNA was present in murine testis and immunohistochemical staining confirmed that E4F1 was enriched in mature Sertoli cells. We generated a conditional knockout mouse model using *Amh-cre* and *E4f1^flox/flox^* lines to study E4F1 fucntion in Sertoli cells and the results showed that *E4f1* deletion caused a significant reduction in testis size and fertility. Further analyses revealed that meiosis progression and spermiogenesis were normal, however, Sertoli cell proliferation was impaired and germ cell apoptosis was elevated in the testis of *E4f1* conditional knockout mice. On the basis of these findings, we concluded that E4F1 was expressed in murine Sertoli cells and served important functions in regulating Sertoli cell proliferation and fertility.

## 1. Introduction

Spermatogenesis is a complex cellular differentiation process including mitosis of spermatogonia, meiosis of spermatocytes and spermiogenesis. Sertoli cells, which locate within the seminiferous tubules, directly interact with germ cells and supply essential factors for developing spermatogenic cells [1]. Sertoli cells secrete niche factors to promote the maintenance of spermatogonial stem cells [2], produce regulatory factors to control meiosis [3], and provide structural and nutritional supports to direct spermatid development [4]. Defects in Sertoli cell function often cause abnormal spermatogenesis and sterility [5]. From cell ablation studies, it has been recognized that the number of Sertoli cells determines testis size and daily sperm production [6]. Sertoli cells also influence testicular blood vessel architecture and secretion of testosterone and estrogen [7,8,9]. Therefore, revealing new insights into Sertoli cell biology is crucial for understanding animal spermatogenesis.

Sertoli cells are specified from a bipotential somatic precursor cells in early fetal stage through a *Sry* (Y-linked testis-determining gene) and *Sox9* (Sry-box containing gene 9) dependent genetic program [10,11]. After specification, Sertoli cells expand in number rapidly during the fetal and early postnatal periods before gradually enter a terminal differentiated state after puberty [12,13]. Thyroid hormone is the master regulator of Sertoli cell proliferation and maturation in rodents. Neonatal hypothyroidism extend murine Sertoli cell proliferation and a significant increase in Sertoli cell number and sperm production [14]. Thyroid hormone has conserved functions because it also inhibits the mitosis of Sertoli cells in bull [15], pig [16] and other animal species [17]. Follicle stimulating hormone (FSH) and activins stimulate Sertoli cell proliferation [18,19]. Bone morphogenetic protein 7 (BMP7), Interleukin-1, and Insulin growth factor 1 (IGF1) are potent mitogens for Sertoli cells in vitro and conditional deletion of IGF-1R in Sertoli cells caused defects in Sertoli cell proliferation and increased apoptosis [20,21,22]. These hormones and growth factors likely work with cell cycle inhibitors p27kip1, p21Cip1 and Rb1 in Sertoli cells. In the testis of p27 or p21 knockout mice, Sertoli cell number and daily sperm production were significantly increased [23]. Deletion of retinoblastoma protein (Rb1) induced mature Sertoli cells to continue cycling, therefore, caused severe defects in spermatogenesis [24]. Key cell cycle regulators that control Sertoli cell mitosis have been partially elucidated, however, transcription factors that direct Sertoli cell growth and maturation remain largely unknown.

Several transcription factors have been demonstrated to be essential for Sertoli cell proliferation. The major function of Rb1 is to suppress E2F transcription factors and knockout transcription factor E2F3 in Sertoli cells rescued the phenotype in Rb1 conditional knockout animals [25]. Transcription factors upstream stimulatory factor (USF) 1 and USF2 are expression in Sertoli cells and *Usf1* knockout mice showed defects in spermatogenesis [26]. Zinc finger transcription factor kruppel-like factor (Klf) 4 is responsive to FSH stimulation and involved in Sertoli cell maturation and proliferation [27]. Estrogen receptors ESR1 and ESR2 activate CCND1 to modulate Sertoli cell proliferation [28]. Hyopoxia indicule factors (HIFs) are regulated by FSH and likely play roles in Sertoli cell proliferation [29]. Among these transcription regulators, Rb1-E2F3 system is the decisive factor determining Sertoli cell proliferation [25], therefore, identifying and elucidating functional roles of factors in the Rb1-E2f regulatory network may help expand the list of transcription factors in the regulation of Sertoli cell function.

Transcription factor E4F1, originally identified as a regulator of the viral E4 and E1A promoters [30,31], interacts with Rb1 and plays crucial roles in cell proliferation and stem cell fate decisions [32,33,34,35]. *E4f1* deficient embryos die at the peri-implantation stage due to defects in mitotic progression, chromosomal segregation and apoptosis [33]. In quiescent cells, E4F1 binds to hypophosphorylated Rb1 to maintain cell cycle arrest [36]. In line with this observation, overexpression of E4F1 in fibroblasts suppresses the progression from G1 to S phase [37]. E4F1 controls cyclin A expression by repressing its promoter activity [34]. In hematopoietic stem and progenitor cells, E4F1 directly interacts with the checkpoint kinase 1 (CHK1) to regulate cell cycle progression and apoptosis [38]. Recent studies suggest that in addition to its role in cell proliferation, E4F1is a potent regulator of pyruvate and lactate metabolism [39]. Despite these important findings, the functional role of E4F1 in Sertoli cell has not been determined.

Because the proliferation of Sertoli cells is tightly controlled and E4F1 is a key regulator of cell cycle progression, we hypothesized that E4F1 served crucial roles in Sertoli cells. Using a conditional mouse model, the present study showed that E4F1 expression was enriched in Sertoli cells and loss of *E4f1* in Sertoli cells led to reduced Sertoli cell number and testis size. Fertility of *E4f1* conditional knockout animals was impaired and Sertoli cells lacking E4F1 activity exhibited reduced mitotic index. Together, these findings indicate that transcription factor E4F1 is expressed in murine Sertoli cells and crucial for Sertoli cell mitotic progression and fertility.

## 2. Materials and Methods

### 2.1. Animals

All animal procedures were conducted in accordance with the Guide for the Care and Use of Laboratory Animals and were approved by the Animal Welfare and Ethic Committee at the Northwest Institute, Chinese Academy of Sciences. *Amh-Cre* (JAX Stock No. 007915) mice were mated with *E4f1^flox/flox^* line (Dr. Guy Sauvageau laboratory, University of Montreal, Montreal, Canada) [38] to generate *Amh-Cre; E4f1^flox/+^* male mice. *Amh-Cre; E4f1^flox/+^* male mice were mated with *E4f1^flox/flox^* to generate *Amh-Cre*; *E4f1^flox/flox^* (designated hereafter as *E4f1* cKO) male mice. *Amh-Cre*; *E4f1^flox/+^* littermates were used as controls. All mice were maintained on a mixed 129S2/SvPasCrl; FVB/N genetic background.

### 2.2. Fertility Test and Sperm Concentration Analysis

45 days old control or E4f1 cKO males were paired with adult wildtype female mice. One male was mated with three females for 3 months. Seven control and seven E4f1 cKO animals were used in the fertility test. Average litter size was recorded to assess fertility. For sperm count, epididymis was put in 1 mL in HEPES medium and shred to release sperm. Then we used hemocytometer to count sperm.

### 2.3. Histological Analysis

Testes were fixed in Bouin’s solution. After dehydration, tissues were embedded with paraffin (HistoCore Arcadia, Leica, Mannheim, Germany). Paraffin-embedded tissues were then cut for 5 µm by microtome (Leica RM2235, Leica, Mannheim, Germany). After rehydration, sections were stained with hematoxylin and eosin (H&E). Images were examined by using a microscope (ECLIPSE E200, Nikon, Tokyo, Japan), and captured by CCD (MS60, MshOt, Guangzhou, China).

### 2.4. Meiosis Analysis

Meiosis analysis was conducted as described previously [40]. Briefly, the tunica albuginea was removed to release the seminiferous tubules into a hypotonic extraction buffer containing 30 mM Tris, 50 mM sucrose, 17 mM sodium citrate dehydrate, 5 mM EDTA, 0.5 mM DTT and 0.5 mM Pheylmethylsulfonyl fluoride (PMSF), pH 8.2–8.4 (pH set by using boric acid) for 20 min. Subsequently, a few seminiferous tubules were placed into 60 µL 100 mM sucrose and torn to pieces to release the cell using two 1 mL syringe needle. Then 20 µL cell suspension was overspread on adhesive slides that were dipped in 1% paraformaldehyde, pH 9.2(pH set by using boric acid), containing 0.15% Triton X-100 in ddH_2_O. The slides were stored in a hot humid chamber overnight. The slides were then dried in the air after dipped in primary antibody dilution buffer solution (ADB) (1% bull serum albumin (BSA), 0.1% cold fish skin gelatin, 0.5% Triton X-100 in 0.01 M phosphate buffered saline (PBS)). 60 µL diluted antibody was placed onto the slides, sealed with glass coverslips, and put the slides into a humid chamber overnight at 37 °C. Next, we removed coverslips and immersed the slides into ADB for 1 h. The secondary antibody was diluted in ADB and placed 60 µL of the diluted antibody onto the slides. After sealed with a glass coverslip, the slides were put into a humid chamber overnight at 37 °C and washed in PBS for 1h. Finally, Hoechst33342 were added for 1 min and the slides mounted in 50% glycerol before examining under a microscope (Leica).

### 2.5. Immunohistochemistry (IHC) and Immunofluorescence (IF)

Testes were fixed in 4% paraformaldehyde (PFA) for immunohistochemistry and immunofluorescence. Sections were boiled in 10 mM sodium citrate (pH 6.0) for about 20 min for antigen retrieval and IHC for E4F1 expression was blocked with endogenous peroxidase with 3% H_2_O_2_. PBS washed sections for 5 min three times. The sections were incubated in the 10% blocking serum for 1 h at room temperature and incubated with primary antibodies overnight at 4 °C. Normal IgG was used for negative controls. After washed in PBS for three times (10 min each), the sections were incubated with secondary antibodies for 1 h at room temperature. The E4F1 expression was visualized with 3,3′-diaminobenzidine tetrachloride (DAB) solution. For detection of immunofluorescent signal, slides were added Hoechst33342 for 1 min, then wash in PBS. Digital images were captured with a microscope (Leica, Mannheim, Germany). Primary antibodies were listed in Supplementary Table S1.

### 2.6. TUNEL Labeling

To determine the number of apoptotic cells, the sections of testis were processed for terminal deoxynucleotidy1 transferase-mediated dUPT nick end labeling (TUNEL) using a TUNEL staining kit (Beyotime, Shanghai, China) according to the manufacturer’s protocol. Co-Immunofluorescent staining of SOX9 and TUNEL was performed using In Situ Cell Death Detection Kit, POD (Roche, Mannheim, Germany). After the sections were incubated in the 10% blocking serum for 1h at room temperature, SOX9 antibody was added to labeling mixture of In Situ Cell Death Detection Kit, incubated 1 h at 37 °C, and washed in PBS 10 min for three times, and then incubated with 555-conjugated donkey anti-rabbit IgG for 1 h at room temperature. Hoechst33342 incubated the sections for 1 min. Digital images were captured with a microscope (Leica, Mannheim, Germany).

### 2.7. 5-ethynyl-2′-deoxyuridine (EdU) Assay

EdU (RIBOBIO, Guangzhou, China) treated mice at a dosage of 50 mg/kg body weight throught intraperitoneal injections. Two hours after EdU injections, testes were collected. Before EdU detection, immunofluorescent staining of TRA98 was carried out. Then EdU was detected according to the manufacturer’s instructions of Cell LightTM EdU Apollo 567 in vivo Kit (RIBOBIO, Guangzhou, China).

### 2.8. qRT-PCR Analysis

Total RNA was purified from mice testes by the Trizol method (Ambion, Austin, TX, USA). cDNA was synthesized using StarScript II First-strand cDNA Synthesis Mix With gDNA Remover Kit (GenStar, Guangzhou, China). The qRT-PCR analysis was performed on the ABI ViiA7 Real-time PCR System (Applied Biosystems, Foster City, CA, USA) with SYBR Green master mix (Genstar, Guangzhou, China), and *Gapdh* was used as an internal control. Primer sequences used for qRT-PCR assay are: *E4f1* forward: CCAGATGAACCCATCACT; *E4f1* reverse: TGCCCACTTCCAACAA; *Gapdh* forward: AGGTCGGTGTGAACGGATTTG; *Gapdh* reverse: TGTAGACCATGTAGTTGAGGTCA; *p21* forward: GCAGATCCACAGCGATATCC; *p21* reverse: CAACTGCTCACTGTCCACGG; *Rb1* forward: CTTGAACCTGCTTGTCCTCTC; *Rb1* reverse: GGCTGCTTGTGTCTCTGTATT

### 2.9. Statistical Analysis

Data are presented as means ± s.e.m. for more than three independent experiments and at least three animals were used for each genotype. Two hundred (200) spermatocytes were used to analyze process of meiosis and 100 seminiferous tubules were used for diameter measurement. The average number of tubules used to analyze Sertoli cell number per cord was 22 per mouse. The average number of Sertoli cells used to analyze germ cells number per Sertoli cell was 1000 per mouse. The number of cords used to analyze the percentage of EdU^+^ cells in SOX9^+^ cells was 20. The number of Sertoli cells used to quantify percentage of TUNEL^+^ Sertoli cells was 2000 for each genotype. And 3 sections were used for each animal. Differences between means were examined using the t-test function of GraphPad Prism 5 (GraphPad Software Inc., La Jolla, San Diego, CA, USA). Differences between means were considered significant at *p* < 0.05.

## 3. Results

### 3.1. The Relative mRNA Expression and Protein Localization of E4f1 in Postnatal Mouse Testes

**Figure 1:**
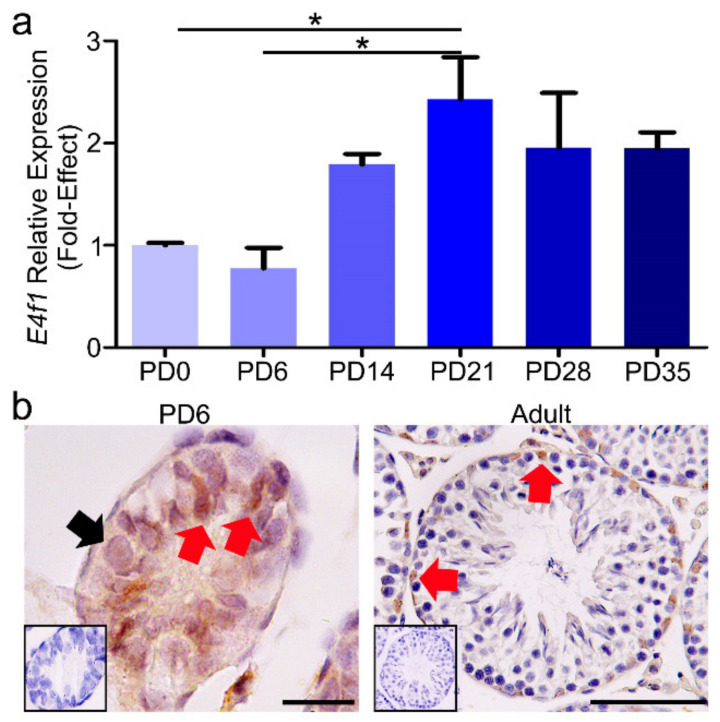
The relative mRNA expression and protein localization of E4F1 in postnatal mouse testes. (**a**) Quantification of mRNA expression of *E4f1* in testes of mice at different development stages. Data were analyzed by mean ± SEM for 3 mice per stage. * denote significantly at *p* < 0.05. (**b**) Immunohistochemical staining of E4F1 in testes at PD6 (scale bar = 20 µm) and adult (scale bar = 1 00 µm) male mice. Black arrow indicates spermatogonia and red arrow indicates Sertoli cells.

In mice, Sertoli cells proliferate in fetal and neonatal periods of development and enter cell cycle quiescence around postnatal day (PD) 12 to 16 [13,41]. Sertoli cells mature to support the development of round spermatid by PD21 [42]. Firstly, we measured relative expression of *E4f1* mRNA in testes of mice at PD0, PD6, PD14, PD21, PD28, and PD35 using qRT-PCR. The results showed that expression of *E4f1* transcript was significantly up-regulated at PD21 (*p* < 0.05) (Figure 1a). Next, we examined the expression and cellular localization of E4F1 in testis using IHC. Negative control did not generate staining signal while E4F1 antibody showed immunoreactive signal in Sertoli cells and spermatogonia at PD6. Interestingly, we detected strong E4F1 staining in Sertoli cells at PD35 (Figure 1b). Together, these data suggested that E4F1was expressed in murine testis and enriched in mature Sertoli cells.

### 3.2. E4f1 Deletion in Sertoli Cells Leads Reduced Testis Size and Male Subfertility

**Figure 2:**
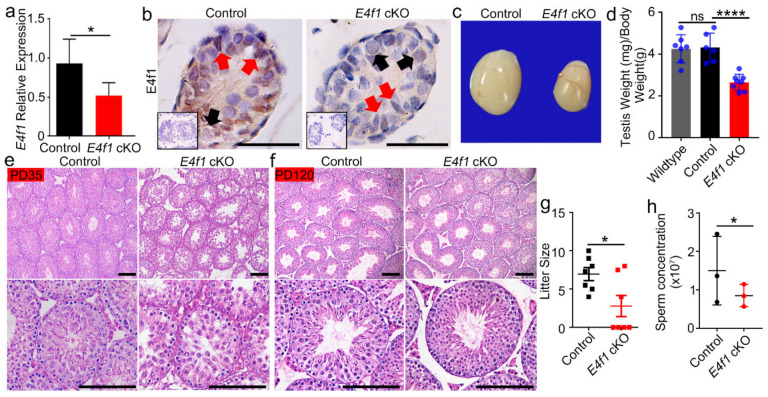
Loss of *E4f1* in Sertoli cells resulted in reduced testis size and Subfertility (**a**) *E4f1* expression level in control and *E4f1* cKO testes. n = 3 (**b**) Reprehensive images of immunohistochemistry staining for E4F1 in control and *E4f1* cKO testes. Black arrow indicates spermatogonia and red arrow indicates Sertoli cells. Scale bar = 50 µm. (**c**) Representative images of the testes from control and E4f1 cKO mice at PD120. (**d**) Testis/Body weight ratio of wildtype (n = 7), control (n = 6) and *E4f1* cKO (n = 8) mice at PD120. (**e**,**f**) Representative images of hematoxylin and eosin (H&E) stained testes of control and *E4f1* cKO male mice at PD35 and PD120. Scale bar = 100µm. (**g**) Quantification of litter size per mice of control and *E4f1* cKO male mice. n = 7. (**h**) Quantification of sperm concentration per mice of control and E4f1 cKO male mice. n = 3. *, **** denote significantly different at *p* < 0.05 and *p* < 0.0001.

We tested whether loss of E4F1 activity influenced Sertoli cell function by generating *E4f1* Sertoli cell conditional knockout mice using Cre-Loxp methodology. Because *Amh-cre* is activated around embryonic day 14.5 [43], *E4f1* was deleted in fetal Sertoli cells. At PD 0, *E4f1* mRNA was significant decreased in testes of *E4f1* cKO compared with that of littermate controls (Figure 2a). IHC staining confirmed that E4F1 was successfully deleted in Sertoli cells because in the testis of *E4f1* cKO animals, spermatogonia maintained E4F1 level while Sertoli cells lost the positive signal (Figure 2b). These data indicated that E4f1 was conditionally deleted and this model was useful to study the effect of E4F1 loss in Sertoli cells.

*E4f1* cKO mice testes were significantly smaller than testes of controls at PD120 (Figure 2c). The testis-to-body weight ratio was reduced by 64% in adult *E4f1* cKO animals (Figure 2d). Histological analysis revealed that the first round spermatogenesis was delayed in the cKO mice at PD35 because elongating spermatids were detected in control animals, however, the most advanced germ cells were round spermatids in *E4f1* cKO animals (Figure 2e). Spermatogenesis recovered in *E4f1* cKO mice at PD120 (Figure 2f). Fertility test using wild-type female mice revealed that the average litter size were 6.971 ± 0.8357 for control and 2.786 ± 1.397 for *E4f1* cKO (n = 7) (Figure 2g). Sperm concentration of *E4f1* cKO mice was reduced compared with that of control (8.5 × 10^7^ vs. 14.93 × 10^7^) (Figure 2h). Together, these findings demonstrated that *E4f1* in Sertoli cells was essential for testis growth and normal fertility.

### 3.3. E4f1 Deletion in Sertoli Cells Did Not Cause Defects in Meiosis and Spermiogenesis

**Figure 3:**
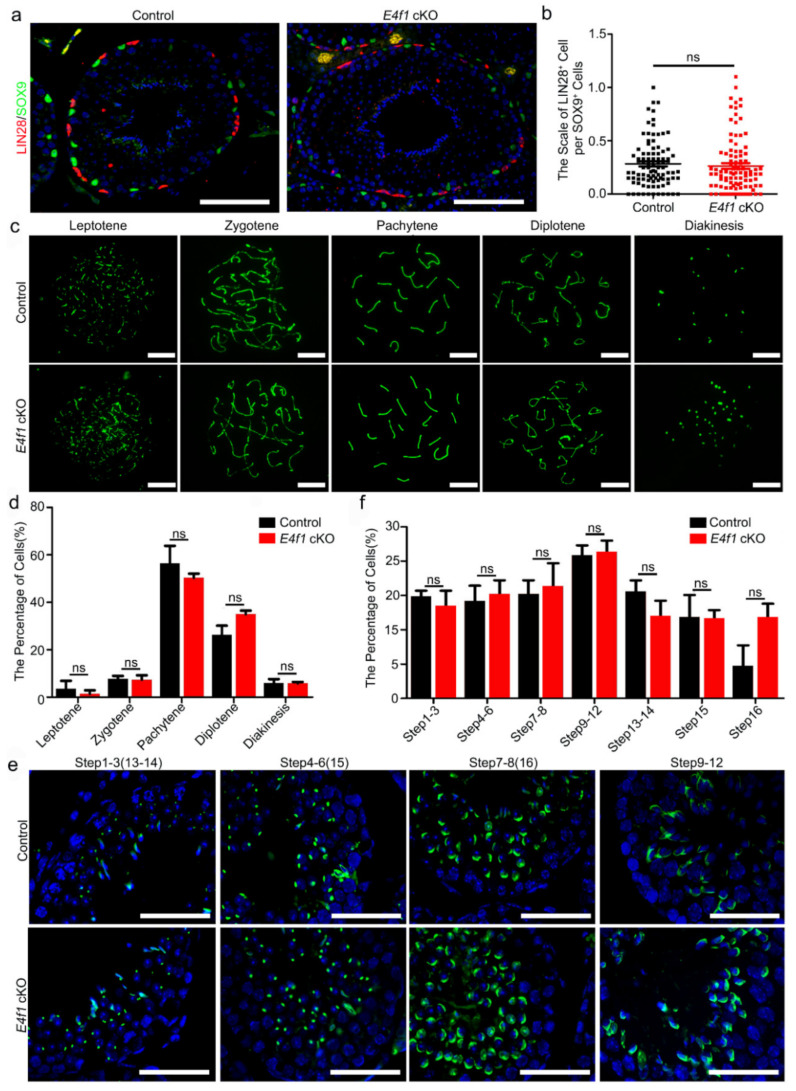
*E4f1* deletion in Sertoli cells did not affect meiosis progression and spermiogenesis (**a**) Immunofluorescent staining for SOX9 and LIN28 in cross-sections of testes from controls and *E4f1* cKO male mice. Scale bar = 100 µm. (**b**) Quantification of the number of spermatogonia per Sertoli cell in testes from controls and *E4f1* cKO male mice. n = 3. (**c**) Immunofluorescent staining for SYCP3 in spread spermatocyte from controls and *E4f1* cKO male mice. Scale bar = 10 µm. (**d**) Quantification of the number of different stages spermatocyte of meiosis per mice of controls and *E4f1* cKO male mice. n = 3. (**e**) Immunofluorescent staining for PNA in cross-sections of testes from controls and *E4f1* cKO male mice. Scale bar = 50 µm. (**f**) Quantification of the number of different stages spermatozoa of meiosis per mice of control and *E4f1* cKO male mice. n = 3.

We then aimed to determine the cause of subfertility in male *E4f1* cKO mice. Because spermatogenesis involves three major developmental phases—spermatogonia proliferation, spermatocytes meiosis and spermiogenesis [5]—we first examined whether the undifferentiated spermatogonial population which contains SSCs was affected. Sertoli cells were labeled with SOX9 and undifferentiated spermatogonia were marked with LIN28 expression. The results showed that one Sertoli cell supported 0.2828 ± 0.023 LIN28^+^ spermatogonia in control and the number was 0.2627 ± 0.02608 in *E4f1* cKO testis, indicating the undifferentiated spermatogonia population was not affected by *E4f1* deletion in Sertoli cells (Figure 3a,b).

We next investigated the meiosis progression using SYCP3 staining as previously described [41]. The results showed that percentages of meiotic cells at leptotene, zygotene, pachytene, diplotene and diakinesis stages did not differ between control and *E4f1* cKO (Figure 3c,d). Finally, we conducted PNA labeling, which recognizes acrosome to examine the morphology of spermatids, and the results indicated the steps of spermatid was not affected by *E4f1* knockout (Figure 3e,f). Because blood-testis-barrier (BTB) was crucial for spermatogenesis, we examined the BTB integrity of control and *E4f1* cKO testis and did not detect any difference (Supplementary Figure S1). Collectively, these findings suggested that the deletion of *E4f1* in Sertoli cells did affect the process of meiosis and spermiogenesis in adult mice.

### 3.4. E4f1 Deletion Resulted in a Reduction in Sertoli Cell Number

**Figure 4:**
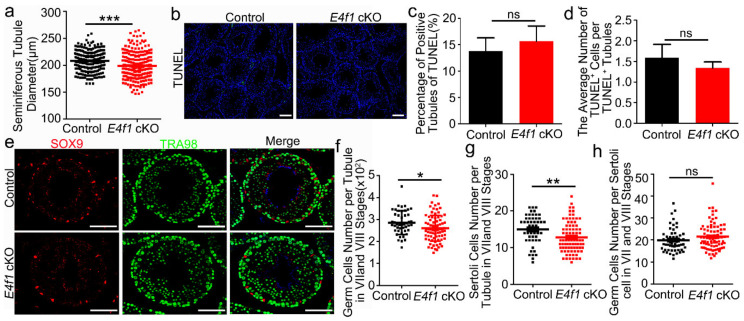
Loss of *E4f1* resulted in decreased Sertoli cell population. (**a**) Quantification of seminiferous tubules diameter per tubule of controls and *E4f1* cKO male mice. n = 3. (**b**) Immunofluorescence staining for TUNEL in cross-section from controls and *E4f1* cKO male mice. Scale bar = 100 µm. (**c**) The percentage of TUNEL positive tubules in testes of controls and *E4f1* cKO male mice. n = 3. (**d**) Quantification of TUNEL positive cells per TUNEL positive tubules in testes of controls and *E4f1* cKO male mice. n = 3. (**e**) Immunofluorescence staining for SOX9 and TRA98 in cross-sections of testes from controls and *E4f1* cKO male mice. Scale bar = 100 µm. (**f**) Quantification of germ cells per tubule in VII and VIII stages of the testes from controls and *E4f1* cKO male mice. n = 3. (**g**) Quantification of Sertoli cells per cord in VII and VIII stages of testes from controls and *E4f1* cKO male mice. n = 3. (**h**) Quantification of germ cells per Sertoli cell in VII and VIII stages of the testes from controls and *E4f1* cKO male mice. n = 3. *, ** and *** denotes significantly different at *p* < 0.05, *p* < 0.01, and *p* < 0.001.

Because *E4f1* deletion in Sertoli cells did not cause severe phenotype in meiosis and spermeiogenesis, we hypothesized that the quantity of spermatogenesis may be affected. To this end, we measured the diameter of seminiferous tubules in testes of control and *E4f1* cKO mice at PD 120. An average of 84 tubules were measured for each control or *E4f1* cKO animal and the results showed that the tubule diameter in testes of *E4f1* cKO mice was significantly decreased (Figure 4a). To rule out the possibility that a global elevation of apoptosis caused germ cell loss, we analyzed the number of apoptotic cells in testicular across-sections of *E4f1* cKO and control mice by using TUNEL staining (Figure 4b). Interestingly, neither percentage of seminiferous tubules containing TUNEL^+^ cells nor the number of TUNEL^+^ cells per seminiferous tubule differ between control and cKO testes (Figure 4c,d). These data indicated that the overall germ cell number was reduced by *E4f1* deletion in Sertoli cells in adult testes.

To validate this observation, we quantified the numbers of Sertoli cell, germ cell and germ cell/Sertoli cell ratio. Germ cells were identified by TRA98 expression and Sertoli cells were marked by SOX9 staining. Because seminiferous tubules at VII and VIII stages contain all types of spermatogenetic cells [12], we measured germ cell in cross-sections of stage VII and VIII tubules. As expected, total germ cell population was decreased in testes of *E4f1* cKO mice because 286.2 ± 7.947 germ cells were found per tubule in control animals and this number was decreased to 261.2 ± 6.515 in *E4f1* cKO animals (Figure 4f). The number of Sertoli cells per stage VII-VIII seminiferous tubule in *E4f1* cKO mice was significantly less than that of controls (Figure 4g). To our surprise, the number of germ cells per Sertoli cell was comparable between control and *E4f1* cKO mice (Figure 4h). Together, we concluded that *E4f1* conditional deletion resulted in a smaller population of Sertoli cells without affecting the ability of Sertoli cells to support spermatogenic cells.

### 3.5. E4f1 Deletion Affected Sertoli Cell Proliferation and Apoptosis during Neonatal Development

**Figure 5:**
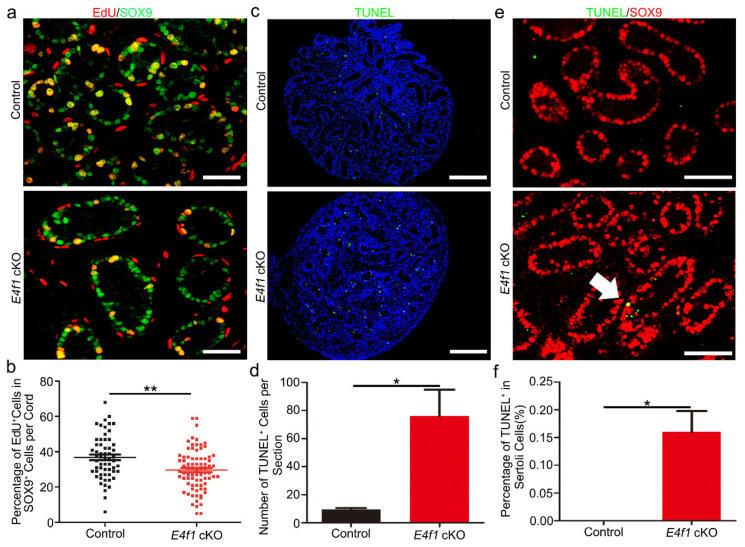
*E4f1* deletion affected proliferation and apoptosis in Sertoli cells. (**a**) Immunofluorescence staining of EdU and SOX9 in cross-sections of testes from control and *E4f1* cKO males at PD0. Scale bar = 50 µm. (**b**) Percentages of proliferative Serotli cells in cross-sections of testes from control and *E4f1* cKO males at PD0. n = 3. (**c**) TUNEL staining in cross-sections of testes from control and *E4f1* cKO males at PD0. Scale bar = 50 µm. (**d**) Quantification of TUNEL^+^ cells in control and *E4f1* cKO males. n = 3. (**e**) Immunofluorescence co-staining of TUNEL and SOX9 in cross-sections of testes from control and *E4f1* cKO males at PD0. Scale bar = 50 µm. (**f**) Quantification of apoptotic Sertoli cells in testes from PD0 controls and *E4f1* cKO male mice. n = 3. * and ** denotes significantly different at *p* < 0.05 and *p* < 0.01.

Because the size of Sertoli cell population is determined by rate and length of proliferation during fetal and neonatal period of development [13], we examined Sertoli cell mitosis at PD0 using EdU assay. 2 h after EdU injection, Sertoli cells were identified by SOX9 expression and cells in S phase of the cell cycle were recognized by EdU staining (Figure 5a). We found a marked decrease in the percentage of EdU positive Sertoli cells in testis of *E4f1* cKO animals (Figure 5b).

Decreased proliferation of cells lacking *E4f1* is coupled to apoptosis [38], we conducted TUNEL assay to evaluate apoptosis in cross-sections of control and *E4f1* cKO testes. It appeared that number of apoptotic cells was increased in *E4f1* cKO testes (Figure 5c,d), we then conducted co-staining of TUNEL and SOX9 to quantify apoptotic Sertoli cells. Sertoli cells in testis of control animals rarely showed signal of apoptosis, however, *E4f1* deletion caused an increase in Sertoli cell apoptosis at PD0 (Figure 5e,f). These findings suggest that loss-of-*E4f1* function in Sertoli cells resulted in defects in proliferation and cell survival.

To investigate underlying cause of impaired cell cycle, we analyzed the mRNA levels of cell cycle related genes *p21* and *Rb1* and pyruvate/lactate gene *Dlat*, and the results showed E4F1 deleted in Sertoli cells did not influence *p21*, *Rb1* or *Dlat* expression (Supplementary Figure S2a,b). We performed co-staining of gH2AX and SOX9, p53 and GATA4, and found that *E4f1* deleted resulted in a increased in p53 protein in Sertoli cells (Supplementary Figure S2a,b). The results showed that impaired cell cycle progression of Sertoli cells maybe related with p53.

## 4. Discussion

Sertoli cells serve central roles in supporting spermatogenesis and defects in Sertoli cell lineage specification and development cause problems in fertility in human and animals [1,12]. Because Sertoli cells only proliferate during fetal and neonatal periods of development, the size of Sertoli cell pool is determined by early puberty [44]. In this study, we showed that transcription factor E4F1 was enriched in murine Sertoli cells and played an important role in regulating Sertoli cell number and fertility.

Sertoli cells directly interact with germ cells and support different types of germ cells in the neonatal or adult testis. Sertoli cells participates all aspects of germ cell development in fetal and postnatal testes. Sertoli cells secret growth factors and cytokine to promote prospermatogonia to spermatogonia transition [45]. Sertoli cells are major contributor of spermatogonial stem cell niche [46]. In adult murine testis, spermatocytes and spermatids are quickly lost upon Sertoli cell removal [47]. In the present study, we found that E4F1 deletion in Sertoli cells affected testis size, however, meiosis progression was normal and spermeiogenesis was not affected. We concluded that reduced fertility in *E4f1* cKO animals was due to decreased sperm production, which is directly caused by a reduction in Sertoli cell population. This conclusion is further supported by the fact that average number of germ cells supported by one Sertoli cell was comparable between control and the conditional knockout animals. A similar phenotype is observed in FSH-deficient male mice. Fsh beta gene knockout males had smaller testis and reduced Sertoli cell number, however, they produce viable sperm and fertile [48]. E4F1 expression is enriched in mature Sertoli cells but did not play a significant role in regulating the quality of spermatogenesis.

Sertoli cell proliferation is regulated by hormones, growth factors and cytokines during fetal and neonatal period of development. FSH stimulates Sertoli cell proliferation by activating cAMP/PKA/ERK1/2 and P13K/Akt/mTORC1 dependent-pathway [49]. Relaxin increases the levels of proliferating cell nuclear antigen (PCNA) in Sertoli cell cultures by activating P13K/Akt and ERK1/2 pathway [50]. And there is crosstalk between FSH and relaxin at the end of the proliferative stage in rat Sertoli cells [35]. Transcription factor c-Myc may have a role in FSH-dependent regulatory network [51]. FSH induces the expression of transcription factor klf4, however, knockout experiments revealed that Klf4 is dispensable for Sertoli cell proliferation [52]. In cultured human smooth muscle cells, E4F1 expression is strongly induced by estrogen and it is recognized as an estrogen-responsive genes that control cell proliferation [53]. It is unclear if a similar mechanism exists in testis, however, we can speculate that E4F1 works as a key transcription factor for FSH, estrogen and other factors involving Sertoli cell proliferation. The upstream factors and signaling pathways that induce E4F1 expression in Sertoli cells should examined in the future studies.

The action of E4F1 is likely independent of Rb1 in Sertoli cells. Rb1 Knockout in Sertoli cells causes severe defects in spermatogenesis and Rb1-decient Sertoli cells reenter cell cycle and undergo dedifferentiation [54]. The phenotype caused by Rb1 inactivation in Sertoli cells can be rescued by E2F3 knockout [25]. Transcription factor ARID4A is an Rb1 binding partner and together, these two proteins function to maintain BTB function [55]. In the present study, deletion of E4F1 did not change BTB integrity. E4F1 regulates lactate metabolism in skeleton muscle cells [56] and one of the major function of Sertoli cells is to supply lactate to developing germ cells [57], we hypothesized that spermatids might be affected by *E4f1* inactivation in Sertoli cells. However, developing of meiotic and postmeiotic germ cells appeared to be normal in the *E4f1* conditional knockout animals. *E4f1* inactivation in Sertoli cells may induce a redirection of the glycolytic flux towards lactate production and secretion, therefore did not affect spermiogenesis. From these data, we concluded that E4F1 is not required for Sertoli cell maturation and terminal differentiation.

Instead, E4F1 works as an important factor controlling Sertoli cell proliferation in neonatal testis. E4F1 interacts with CHK1 and cell cycle arrest caused by E4F1 deletion can be rescued by Chek1 overexpression [38]. In preimplantation embryo, E4F1 promotes cell cycle progression and maintains genome integrity [33]. Cancer cells lacking E4F1 is arrested in G2/M of cell cycle [58]. In Sertoli cells, inactivation of E4F1 impaired G1-S transition and increased apoptosis, however, these cells were not completely arrested. These data suggest that E4F1 function is important but not essential for Sertoli cell mitosis and survival. Other transcription factors that determine Sertoli cells proliferation and function in neonatal testis remain to be identified.

## 5. Conclusions

In summary, we found that E4F1 is expressed in Sertoli cells and spermatogonia of postnatal murine testis and enriched in mature Sertoli cells. Loss of function experiment revealed that E4F1 was not required for maintaining cell cycle arrest or Sertoli cell maturation, however, it played a vital role in the regulation of Sertoli cell proliferation and determining testis size. Collectively, this study provides an important piece of information to our understanding of molecular mechanisms regulating Sertoli cell proliferation. The knowledge gained from this study may be applicable to understand the mechanism of Sertoli cell proliferation in large animals.

## Supplementary Materials

The following are available online at https://www.mdpi.com/2076-2615/10/9/1691/s1, Figure S1: Functional assessment of the BTB in control and E4f1 cKO mice testis, Figure S2: *Rb1*, *p21*, and *Dlat* expression level in PD0 control and E4f1 cKO testes, and γH2AX and SOX9, GATA4 and P53 protein expression in Sertoli cell from PD0 control and E4f1 cKO testes. Table S1: Information of antibodies used in the study.
