# Supplementary material for: E4 Transcription Factor 1 (E4F1) Regulates Sertoli Cell Proliferation and Fertility in Mice
Source: Animals (Basel). 2020 Sep 18;10(9):1691. doi: 10.3390/ani10091691 (PMC7552733; doi:10.3390/ani10091691)
Supplement: Supplementary file 1 [file animals-10-01691-s001.pdf]

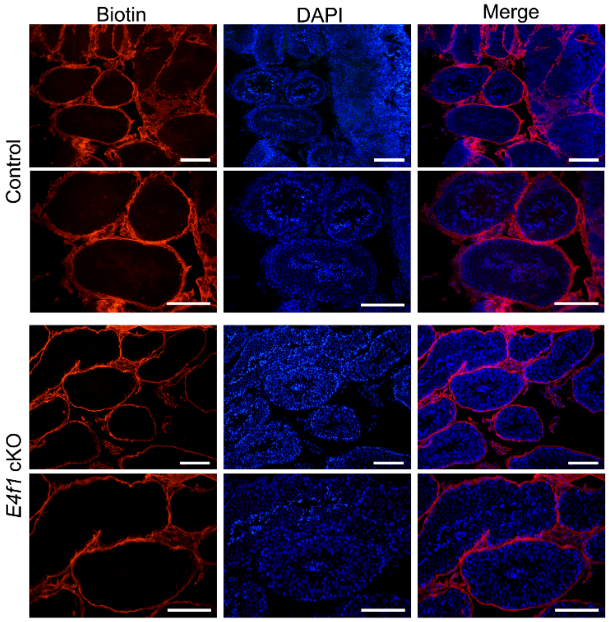

Supplemental Figure S1 Functional assessment of the BTB in control and E4f1 cKO mice testis. Scale bar = 100μm.

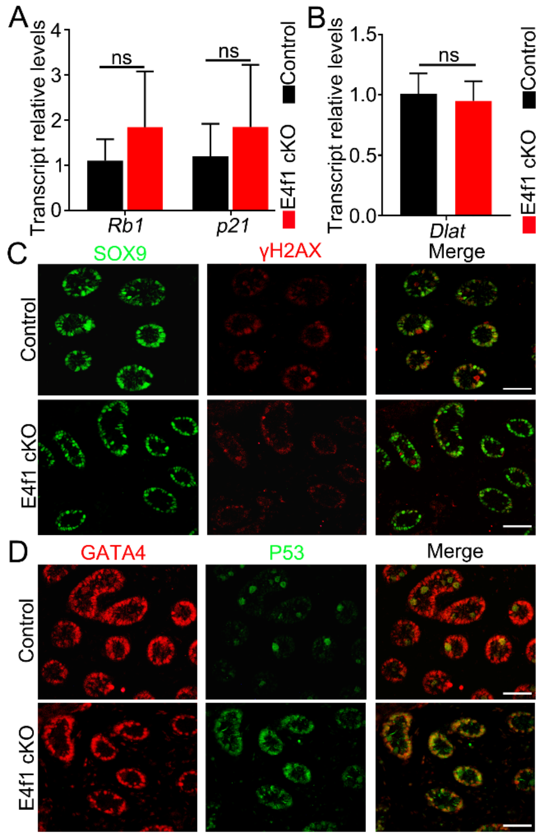

Supplemental Figure S2 (a) *Rb1* and *p21* expression level in PD0 control (n=10) and *E4f1* cKO (n=11) testes. (b) *Dlat* expression level in PD0 control and *E4f1* cKO testes. n=3. (c)(d) Immunofluorescent staining for γH2AX and SOX9, GATA4 and P53. Scale bar = 50μm.

Table S1 Information of antibodies used in the study.

Antibody	Dilution ratio	Company	Catalogue No.
Rabbit anti-E4F1	1:100	Shay bio	AY-00447R
Goat anti-LIN28A	1:200	R&D Systems	AF3757
Rat anti-TRA98	1:200	Abcam	ab82527
Rabbit anti-SOX9	1:200	Abcam	AB5535
Rabbit anti-Sycp3	1:200	Santa cruz	sc33195
Normal goat IgG	1:1000	Sino Biological Inc	CR2
Normal rabbit IgG	1:1000	Cell Signaling Technology	2729s
Normal rat IgG	1:1000	Santa Cruze	sc-2026
